# Mrs. Gladstone's Home in Epping Forest

**Published:** 1889-07-13

**Authors:** 


### Mrs. Gladstone's Home in Epping Forest.

By a Medical Superintendent.

It is always profitable to take a retrospective glance at the origin and history of our curative institutions, especially when they inaugurate, as in the case of convalescent homes, a new departure from the stereotyped forms of medical relief. It is now fifty years since the Metropolitan Convalescent Institution at Walton was established, and during the interval which has elapsed homes of rest for the weak and suffering have multiplied in an increasing ratio both in the country and at the seaside, and have proved an ineffable boon to the multitude recovering from attacks of disease, and to the hospitals by supplementing their work with the best of all restoratives. For many years the Metropolitan Asylum, as it was then called, stood alone as the pioneer of the movement. It was established on the time-honoured principle that each claimant for its privileges should possess a subscriber's recommendation and a doctor's certificate; and although to the initiated these were not always difficult to obtain, there were thousands discharged from hospitals, or who had recovered from illness at their own homes, who were compelled to do without them. It is true that here and there cottage homes were instituted mainly by private benevolence, where, by paying a few shillings weekly patients were boarded and lodged, but it was not till the year 1866, when the cholera devastated the East End of London, that the want of a free home for convalescents from the malady was more acutely felt. Mrs. Gladstone, who, during the epidemic, had been doing good work in the wards of the London Hospital and in other parts of the East End, was alarmed at the fact that many patients who had apparently recovered from cholera relapsed after a while into serious illness at their own homes, from want of food, fresh air, and comfortable surroundings. With the assistance of a few friends, she opened two houses at Upper Clapton for men and women patients. The advantages attending the free and ready admission of the poor to this establishment became so pronounced that, although the cholera disappeared from our midst in a much shorter time than it had ever done before, the home was so greedily taken advantage of by other convalescents that Mrs. Gladstone's committee considered it most desirable, not only to extend the accommodation, but to remove further afield to more salubrious quarters. An opportunity occurring of purchasing, on favourable terms, the freehold mansion and grounds of Woodford Hall, in Essex, the committee gladly availed themselves of it, and in 1869 the inmates of the other home were removed there, after it had undergone some necessary alterations. The new home was found capable of accommodating a larger number of convalescents, and more comfortably than the others, the ground floor being utilised for dining, recreation, and administrative purposes, and the upper floors for dormitories, the divisions betwixt the sexes and also the children of tender years being kept strictly apart. The building stands in ample grounds of its own in front and rear, and is in close contiguity with Epping Forest, to which the patients have daily access. Altogether, the position is as cheerful and salubrious and the surroundings as attractive as one could meet with within an easy distance of London. Patients after passing the committee are received on Mondays and Tuesdays. They travel by the Great Eastern Railway to George Lane Station, about half a mile from the home, at the small charge of eightpence, an important consideration for the majority who have seldom sixpence to bless themselves with. There is a donkey on the premises which assists those unable to walk the distance from the station to the home, besides performing other duties in connection with the establishment which usually fall to the lot of donkeys. Having had occasion to send many hundreds of convalescents or would-be convalescents to the home, and having heard—not for the first time—that the place was not altogether what they expected to find it, I was induced to visit it, without giving any formal notice of my intention, and to judge for myself whether there was any substantial grounds for the complaints which had gained currency in more than one quarter. On entering the grounds, I was met by several boy patients at play, whose bright and cheerful demeanour and evident enjoyment spoke happiness and content, and in another part of the grounds were a number of men, mostly resting on the ground, and clustered round one or two others, apparently engaged in chopping firewood. There could be no doubt, from their appearance and the leisurely way in which the work was being done, that all of them had but recently been discharged from an East End hospital, and some of them were still wearing the livery of the accident ward, in the shape of plaster of Paris bandages and crutches.

Inside the building everything appeared in excellent order, the dormitories for men, women, and children were patterns of neatness, cleanliness, and comfort, while the ground floor recreation-rooms were large and airy, that attached to the women's division opening into a large conservatory, not overwell stocked with plants or shrubs. In the leading corridor we came across a plaque in terra-cotta in high relief of Mrs. Gladstone, an excellent likeness, and over it rather a sickly portrait in oil of the G. O. M. On entering the men's recreation-room it was easy to account for the low estimation in which the home is held by many, for, sitting round the fireplace or loafing about in different parts of the room, the inmates indicated anything but a lively appreciation of the privileges they were enjoying. With one or two exceptions, they were miserably clad, seemed listless and apathetic when spoken to, and apparently incapable or unwilling to indulge either in physical or mental exertion. It would be hardly fair to judge the success of an establishment by the appearance or demeanour of its occupants. Nor could it be said that in the case in question they were not worthy of convalescent relief; at the same time one can readily understand how a large contingent of hospital patients would not be attracted by their new associates. The element of caste, which enters so largely into many of our social institutions, has also penetrated to medical charities, and we need hardly be surprised at its finding a place in convalescent homes. But it was, in point of fact, to meet the needs of the poorest class, the residuum, so to speak, of the population, that Mrs. Gladstone's Home was first established, and it cannot be denied, notwithstanding the shabby externals of its occupants, that it has been a harbour of refuge to thousands, and thoroughly deserving of public sympathy and support. We have heard complaints, not at all confined to the patients of this home, that they are required to engage in work not in keeping with the efforts made for their recovery. The practice of employing convalescents, both men and women, to assist in the general domestic routine of the establishment may be dying out in hospitals and poor-law infirmaries, but in convalescent institutions it is almost universal, and we should be sorry to see it discontinued. As a rule, the majority of patients take kindly to the tasks allotted them, but there are drones in every community, great or small, whose influence is likely to infect other members of the family. Much, almost everything, depends on the tact and judgment of the person in charge, usually the matron, not only with respect to the division of labour, but also with the individual fitness of the members of the household to perform it. The best test of good management, whether in a hospital or in a convalescent home, may be estimated by the smallness of the numbers dismissed for irregularities of conduct, and considering the heterogeneous elements of which Mrs. Gladstone's Home is composed, it reflects infinite credit on the lady who for so many years has conducted its domestic economy, that she should so seldom be compelled to have recourse to this unfortunate alternative.

Continuing our enquiries, and knowing from everybody's experience that good feeding constituted the backbone of the convalescent system, we felt naturally anxious to learn something of the commissariat arrangements of the home. We found the bread, beef, mutton, and milk of excellent quality and liberally supplied, while there was no monotony in the character of the daily meals. The chief articles of food were furnished by local purveyors without any direct contract, which accounted for the prices paid being fully 15 per cent. higher than in the bulk of London hospitals, but there is no doubt that the open system of purchase has numerous advantages both of convenience and confidence in the supply not possessed by the other, and is also better adapted for institutions situated in the country. Notwithstanding the comparatively high cost for food, it does not appear to influence unfavourably the annual average cost of the occupied beds in the establishment. In an analysis of the income and expenditure of eighteen convalescent homes, more or less affiliated with the metropolis, and consequently receiving grants from the Hospital Sunday Fund, it was shown in the number of this journal for October last that the average cost of a permanently occupied bed in these establishments, taken collectively, was not less than £46 per annum, whereas the cost of a bed at Mrs. Gladstone's Home did not amount to more than £21. The significant fact is not difficult of explanation, and affords a practical lesson to philanthropists how their exertions may be multiplied by judicious forethought and economy. The secret consists in the fact that all the people engaged in the work give their services for love. There is no office, no paid secretary, no collector, no advertisements of funds urgently needed, nor any of the paraphernalia which are usually thought essential to float a concern whether charitable or mercantile. For the transaction of ordinary business, such as the admission of patients, the committee of the London Hospital have granted the use of their board-room one afternoon weekly, and Mrs. Gladstone's committee express their sense of obligation and warmest thanks to Mr. Nixon, who from the first establishment of the home has afforded them much assistance and encouragement in their work. The annual reports disclose a fair list of subscribers, which, however, might be strengthened to meet the current expenditure, the deficiency of income having to be made up by donations and other vicarious items on which no permanent reliance can be placed. When these means fail, a letter from Mrs. Gladstone to the newspapers is usually followed by an accession of income, and when affairs are at their worst, as happened a few years ago, a public meeting at the Mansion House was the occasion of replenishing the empty exchequer by making the good work known to many who probably hitherto had been ignorant of its existence. One need never despair of support for a charity like this, for it addresses itself to a want in our social system which cannot be met by poor-law relief, or by the principles which guide the administration of the great bulk of our curative agencies.

